# Chlorfenapyr: a new insecticide with novel mode of action can control pyrethroid resistant malaria vectors

**DOI:** 10.1186/1475-2875-10-16

**Published:** 2011-01-25

**Authors:** Kamaraju Raghavendra, Tapan K Barik, Poonam Sharma, Rajendra M Bhatt, Harish C Srivastava, Uragayala Sreehari, Aditya P Dash

**Affiliations:** 1 National Institute of Malaria Research (ICMR), Sector 8, Dwarka, New Delhi- 110 077, India; 2 National Institute of Malaria Research Field Unit, Raipur, Chhattisgarh, India; 3 National Institute of Malaria Research Field Unit, Nadiad, Gujarat, India; 4 World Health Organization, South East Asia Regional Office, Indraprastha Estate, New Delhi-110002 India

## Abstract

**Background:**

Malaria vectors have acquired widespread resistance to many of the currently used insecticides, including synthetic pyrethroids. Hence, there is an urgent need to develop alternative insecticides for effective management of insecticide resistance in malaria vectors. In the present study, chlorfenapyr was evaluated against *Anopheles culicifacies *and *Anopheles stephensi *for its possible use in vector control.

**Methods:**

Efficacy of chlorfenapyr against *An. culicifacies *and *An. stephensi *was assessed using adult bioassay tests. In the laboratory, determination of diagnostic dose, assessment of residual activity on different substrates, cross-resistance pattern with different insecticides and potentiation studies using piperonyl butoxide were undertaken by following standard procedures. Potential cross-resistance patterns were assessed on field populations of *An. culicifacies*.

**Results:**

A dose of 5.0% chlorfenapyr was determined as the diagnostic concentration for assessing susceptibility applying the WHO tube test method in anopheline mosquitoes with 2 h exposure and 48 h holding period. The DDT-resistant/malathion-deltamethrin-susceptible strain of *An. culicifacies *species C showed higher LD50 and LD99 (0.67 and 2.39% respectively) values than the DDT-malathion-deltamethrin susceptible *An. culicifacies *species A (0.41 and 2.0% respectively) and *An. stephensi *strains (0.43 and 2.13% respectively) and there was no statistically significant difference in mortalities among the three mosquito species tested (p > 0.05). Residual activity of chlorfenapyr a.i. of 400 mg/m^2 ^on five fabricated substrates, namely wood, mud, mud+lime, cement and cement + distemper was found to be effective up to 24 weeks against *An. culicifacies *and up to 34 weeks against *An. stephensi*. No cross-resistance to DDT, malathion, bendiocarb and deltamethrin was observed with chlorfenapyr in laboratory-reared strains of *An. stephensi *and field-caught *An. culicifacies. *Potentiation studies demonstrated the antagonistic effect of PBO.

**Conclusion:**

Laboratory studies with susceptible and resistant strains of *An. culicifacies *and *An. stephensi*, coupled with limited field studies with multiple insecticide-resistant *An. culicifacies *have shown that chlorfenapyr can be a suitable insecticide for malaria vector control, in multiple-insecticide-resistant mosquitoes especially in areas with pyrethroid resistant mosquitoes.

## Background

Insecticides belonging to different groups, namely DDT (organochlorine) and malathion (organophosphate) have been in use for the past two to five decades in vector control programmes in India and later synthetic pyrethroids were introduced for indoor residual spraying (IRS) in areas with multiple insecticide-resistant vectors, and for the treatment of mosquito nets (insecticide-treated nets or ITNs) and more recently for manufacturing of long-lasting insecticidal nets (LLINs). Due to continued use of these insecticides/interventions, two major malaria vector species in India, *Anopheles culicifacies *and *Anopheles stephensi *have developed multiple insecticide resistance. Synthetic pyrethroids were introduced in public health programme in 1990s to control DDT-malathion-resistant mosquitoes in some areas. *Anopheles culicifacies*, the major malaria of vector in rural and peri-urban areas of India have shown resistance to pyrethroids (Raghavendra, unpublished data). *Anopheles stephensi, *another major vector of malaria in urban areas, has also developed resistance to DDT, dieldrin and malathion [1].

Since the introduction of pyrethroids in the 1980s, no new adulticides have been approved for vector control by the World Health Organization (WHO) [2]. Thus, alternative insecticides/interventions to control pyrethroid-resistant mosquitoes and prevent further spread of resistance need to be developed. For this reason, insecticides belonging to classes unrelated to the above three groups with different modes of action need to be further investigated for vector control potential. In this endeavor, we have tested the insecticide molecule, chlorfenapyr, a pyrrole class insecticide for its use in vector control.

Chlorfenapyr is used commercially for termite control and crop protection against a variety of insect and mite pests [3-5]. Chlorfenapyr is a pro-insecticide and oxidative removal of the N-ethoxymethyl group of chlorfenapyr by mixed function oxidases leads to a toxic form identified as CL 303268 which functions to uncouple oxidative phosphorylation in the mitochondria, resulting in disruption of ATP production and loss of energy leading to cell dysfunction and subsequent death of the organism. This molecule has low mammalian toxicity and is classified as slightly hazardous insecticide as per WHO criterion [6]. Due to its novel mode of action, chlorfenapyr is unlikely to show any cross resistance to standard neurotoxic insecticides as observed in *Anopheles gambiae *[7], *Anopheles funestus *[8], *Anopheles quadrimaculatus *[9], *Aedes aegypti *[10] and *Culex quinquefasciatus *[7,11].

In the present study, chlorfenapyr was evaluated for its residual efficacy and persistence on different substrates against insecticide susceptible *An. culicifacies *and *An. stephensi *mosquitoes. The cross-resistance patterns were assessed in laboratory-reared strains of multiple insecticide-resistant *An. stephensi *and field collected multiple insecticide-resistant *An. culicifacies *from Chhattisgarh and Gujarat states, India. Potentiation studies were also undertaken with a known mixed function oxidase (MFOs) inhibitor, piperonyl butoxide (PBO) on *An. stephensi *to assess the impact (synergism or antagonism) on the susceptibility to chlorfenapyr.

## Methods

### Mosquito strains

Laboratory reared strains maintained at controlled temperature (27 ± 2°C) and relative humidity (70-80%) with 14:10 h light:dark photoperiod, were used for the studies. The following Indian mosquito strains were used:

#### Laboratory strains

i. *An. culicifacies *species A-Laboratory reared DDT-malathion-deltamethrin-bendiocarb susceptible strain collected from Ghaziabad, Uttar Pradesh, established in 1990

ii. *An. culicifacies *species C-Laboratory reared DDT-resistant strain collected from Jabalpur, Madhya Pradesh, established in 1994

iii. *An. stephensi *Okhla-Laboratory reared DDT-malathion - deltamethrin-susceptible strain collected from Okhla, Delhi in 1977

iv. *An. stephensi *Sonepat-Laboratory reared DDT -malathion - deltamethrin - bendiocarb susceptible strain collected from Sonepat, Haryana in 1996

v. *An. stephensi *Nadiad-Laboratory reared DDT -malathon - deltamethrin-bendiocarb - susceptible strain collected from Nadiad, Gujarat in 2009

vi. *An. stephensi *Goa-field collected DDT-malathion-resistant/deltamethrin-tolerant mosquitoes from Goa and established in 2009

#### Field collected mosquitoes

*(i) An. culicifacies-*DDT-malathion-deltamethrin-bendiocarb-resistant mosquitoes collected from Raipur district, Chhattisgarh state in 2009

*(ii) An. culicifacies-*DDT-malathion-deltamethrin-resistant/bendiocarb-tolerant mosquitoes collected from Panchmahals district, Gujarat state in 2009

*(iii) An. culicifacies-*DDT-malathion-deltamethrin-resistant/bendiocarb-tolerant mosquitoes collected from Vadodara district, Gujarat state in 2009

*An. culicifacies *from Ghaziabad and Jabalpur and *An. stephensi *from Okhla, Delhi were used for the determination of diagnostic dose and persistence studies on different substrates. Laboratory-reared *An. stephensi *from Sonepat and Goa and field-collected *An. culicifacies *from Raipur, Panchmahals and Vadodara districts were used for cross-resistance studies. The wild-caught adult mosquitoes were used for cross-resistance studies. Indoor-resting mosquitoes were collected using aspirator and torch light in the early morning hours in the selected localities and were transferred to the base laboratory in cloth cages for experiments. Potentiation studies were carried on laboratory reared insecticide susceptible strain of *An. stephensi *from Sonepat and resistant strain from Goa.

### Insecticide-impregnated paper

Chlorfenapyr impregnated papers of different concentrations, *viz. *0.25 to 5% (0.25, 0.5, 0.75, 1, 1.5, 2, 2.5, 3, and 5%) and silicone oil control paper were procured from Vector Control Research Unit, University Sans Malaysia, Malaysia for WHO adult susceptibility tests.

### Preparation of substrates

The surfaces were prepared following WHO guidelines [12] with some modifications. Aluminum trays (L-50 cm × W-50 cm × D-2 cm) were fitted with wire mesh at 0.5 cm above the bottom for holding the surfaces. Total thickness of the fabricated surface was 1 cm. Surfaces were dried in sunlight for two days and stored in a cool dark place until insecticide treatment.

#### Different substrates prepared for the study include

a) Mud: Potter's clay mixed with a small quantity of paper and rice husk as binding materials. b) Mud + Lime: to the above prepared substrate a coat of washers lime (CaO) applied and dried. c) Cement: Sand and cement in the ratio of 3:2 mixed with a small quantity of plaster of Paris molded into tray and dried. d) Cement + Distemper paint: to the above prepared cement substrate, a coat of distemper paint was applied twice and allowed to dry. e) Wood: Wooden planks (unpainted wood) (50 cm × 50 cm × 2 cm).

### Impregnation of substrates with insecticide

A compression spray pump of 3.0 L capacity with a flat fan (Imatic-NC F80/12/3, 80°-SF-03 nozzle type) and discharge rate of 800 ml/min was used for spraying on the substrates. Suspensions of chlorfenapyr SC in dosages of 0.25, 0.5, 1, 2, 3, 4, 8 and 16% corresponding to 12.5 to 800 mg/m^2 ^(12.5, 25, 50, 100, 200, 400, 600, 800 mg/m^2^) were prepared and sprayed on the substrates. Substrates were stored in a cool dark place at ambient temperature and humidity conditions until used for cone bioassays.

### Determination of diagnostic dose

Adult susceptibility tests were carried out by challenging 20-25, 3-4 day old sugar-fed laboratory-reared *An. stephensi *and *An. culicifacies *to a range of 0.25 to 5.0% chlorfenapyr -impregnated papers in WHO test kits. Insecticide-susceptible laboratory strains were used for the determination of diagnostic dose. Insecticide-impregnated papers were employed for a maximum of 5 exposures. To standardize exposure time and holding periods, mosquitoes were exposed for 60, 90, 120, 150 and 180 min, and mortality was scored after holding them for 24, 48 and 72 h. Adults were considered dead if they were ataxic. Test mortality was corrected by applying the Abbott's formula [13] when control mortality was between 5 and 20%. Mortality data were subjected to log-probit regression analysis using SPSS v 10.0 [14] and LC50 and LC99 values were calculated. A dose twice the value of LC99 was considered as the diagnostic dose [12] to discriminate the susceptible from resistant ones in the test population.

### Persistence studies

Efficacy of chlorfenapyr-treated substrates was assessed under laboratory conditions using cone bioassays [12]. Four replicate substrates per dose were used for each mosquito species, along with appropriate controls. Four substrates were rotated between consecutive tests, with one substrate being randomly excluded during each rotation. Residual efficacy was determined by challenging laboratory reared, 3-4 day old sugar-fed *An. stephensi *(Okhla, Delhi) and *An. culicifacies *A (Ghaziabad, U.P.). Cone bioassays were carried out on sprayed surfaces on Day 1, Day 3 and at weekly intervals. On each substrate four cones were fixed using elastic tape. Rims of the cones were lined with a thin sponge to avoid opportunistic escape of mosquitoes. Four replicates of 10 female mosquitoes each, were exposed for 30 min to each of the given substrates and doses [12]. After exposure, the mosquitoes were gently transferred into plastic holding cups covered with a net fastened with rubber band. A cotton wool pad soaked in 10% glucose solution was placed on the net for access of sugar solution for the mosquitoes. Mortality counts were made after 24, 48 and 72 h.

### Insecticide susceptibility tests

Insecticide susceptibility tests were conducted to determine the susceptibility status to different insecticides and cross resistance to chlorfenapyr in the following strains: (i) laboratory-reared insecticide-susceptible *An. stephensi *from Sonepat (Haryana state) and Nadiad (Gujarat state); (ii) laboratory-reared multiple insecticide-resistant strain of *An. stephensi *from Goa state; and (iii) Wild-caught multiple insecticide-resistant *An. culicifacies *from Districts Raipur (Chhattisgarh state) and Nadiad (Gujarat state). Mosquitoes were exposed for 1 h to DDT (4%), malathion (5.0%), bendiocarb (0.1%) and deltamethrin (0.05%) and mortality was scored after 24 h holding period and for 2 h exposure to 5.0% chlorfenapyr and mortality was scored after 48 h holding period.

### Potentiation bioassays

Bioassays were carried out with PBO, a known MFO inhibitor. To determine the sub-lethal concentration PBO impregnated papers (12 × 15 cm, Whatman Grade No.1 filter paper) were prepared by evenly applying 2.0 ml solution of a given concentration of PBO in olive oil and acetone [15] on Fakir Board [12], air dried for 24 h and stored in aluminum foil till use. For assessing sub lethal concentration of PBO for synergism/antagonism studies against deltamethrin, mosquitoes were exposed to 5, 10 and 15% impregnated papers for 1 h and held for 24 h, while for chlorfenapyr, mosquitoes were exposed to 5, 10 and 15% PBO impregnated papers for 2 h and held for 48 h. Sub-lethal concentration of PBO was determined for potentiation studies, i.e maximum concentration at which no mortality occurred at the end of the holding periods. A 15% concentration was found to be the sub lethal concentration of PBO and was used for potentiation studies.

Mosquitoes were exposed serially to PBO impregnated papers and to deltamethrin or chlorfenapyr-impregnated papers to demonstrate synergism/antagonism to toxicity of these insecticides using insecticide-susceptible *An. stephensi *(Sonepat strain) and -resistant *An. stephensi *(Goa strain). Bioassays were carried out by pre-exposure to the sub-lethal concentration of PBO (15%)-impregnated papers for 1 h followed by exposure to 0.05% deltamethrin-impregnated papers for 1 h and mosquitoes were held for 24 h to assess synergism/antagonism to deltamethrin toxicity. While exposures were made to sub-lethal concentration of PBO (15%) impregnated paper for 2 h followed by exposure to 5.0% chlorfenapyr-impregnated papers for 2 h and held for 48 h to assess synergism/antagonism to chlorfenapyr toxicity. Appropriate controls were run simultaneously for the experiments and percent mortality was corrected with control mortality by applying the Abbott's formula [13].

## Data analysis

Dose/time mortality data were subjected to log-probit regression analysis [16]. LD50/LT50, LD99/LT90, values were calculated with 95% fiducial limits using SPSS v 10.0 [14]. Further, synergistic/antagonistic indices (RR50 and RR90) were calculated as a ratio between the corresponding LT50 or LT90 values of insecticide alone and PBO + insecticide. Assessment of these outcome variables between treatments relative to control was analysed by regression analysis and analysis of variance (ANOVA) using SPSS 10.0 to test the difference among doses and also among different substrates.

## Results

### Diagnostic dosage of chlorfenapyr

Mosquitoes were exposed to the range of concentrations (0.25-5.0%) of chlorfenapyr-impregnated papers. Complete mortality of mosquitoes in susceptibility test was registered at 2.5% concentration and beyond, hence data are presented for exposures against 0.25-2.5% impregnated papers only. Mosquitoes were exposed to different doses (0.25-2.5%) and combinations of exposure times (60-180 min) and holding periods (24-72 h). Based on the observed mortality data, an exposure time of 2 h followed by holding period of 48 h was found appropriate as these employed periods for exposure and holding registered maximum mortality and were found suitable for determining the diagnostic dose to discriminate susceptible from resistant ones in a population. The DDT-resistant/malathion-deltamethrin susceptible strain of *An. culicifacies *C showed higher LD50 and LD99 (0.67 and 2.39% respectively) values than the DDT-malathion-deltamethrin susceptible *An. culicifacies *A (0.41 and 2.0% respectively) and *An. stephensi *strain (0.43 and 2.13% respectively) (Table 1). However, there was no statistically significant difference in mortalities among the three mosquito species tested (p > 0.05). The LD99 values of chlorfenapyr were 2.0, 2.39 and 2.13% for *An. culicifacies *A, *An. culicifacies *C and *An. stephensi *respectively. Following the standard criterion, double the calculated value of LD99 approximating to 5.0% was considered as the diagnostic dose for determining the susceptibility status in these two species.

**Table 1:** Dose- mortality response of *An. culicifacies species *A and species C and *An. stephensi *against different doses of chlorfenapyr impregnated papers (2 h exposure and 48 h holding)

% Dose	Species		
	*An. culicifacies* species A (Ghaziabad, UP)	*An. culicifacies* species C (Jabalpur, MP)	*An. stephensi * (Okhla, Delhi)
	**% mortality (No. exposed)**		

**0.25**	23.5 (102)	10.0 (100)	21.0 (100)

**0.5**	58.8 (102)	23.3 (180)	ND

**0.75**	86.2 (102)	54.0 (100)	74.0 (100)

**1.0**	88.2 (102)	74.0 (100)	91.2 (105)

**1.5**	96.0 (102)	93.0 (100)	95.0 (100)

**2.0**	99.0 (300)	99.2 (261)	99.5 (199)

**2.5**	100 (101)	100 (60)	ND

	**Lethal doses**		

**LD**_**50**_**(FL)**	0.41 (0.36-0.45)	0.67 (0.56-0.79)	0.43 (0.31-0.54)

**LD**_**99 (FL)**_	2 (1.70-2.48)	2.39 (1.78-3.95)	2.13 (1.57-3.55)

**χ**^**2 **^**(df), P-value**	3.392 (5), 0.640	20.46 (5), 0.001	9.46 (4), 0.05

ND - Not done : FL- Fiducial limits

χ^2 ^= heterogeneity of response, df = degrees of freedom

### Residual efficacy of chlorfenapyr

Efficacy of chlorfenapyr on different substrates spread at 12.5 to 200 mg/m^2 ^has shown a drastic reduction in efficacy within two weeks after spraying. Hence, bioassays were continued on substrates with dosages of a.i, 400 mg/m^2^, 600 mg^2^/mand 800 mg/m^2^. *Anopheles culicifacies *showed 100% mortality in cone bioassays up to 20 weeks and mortality remained >88% up to 24 weeks on all the substrates with 400 mg/m^2 ^and 600 mg/m^2 ^doses, and up to 28 weeks with 800 mg/m^2 ^dose. While *An. stephensi*, registered >80% mortality on all the substrates up to 34 weeks with 400 mg/m^2^, 600 mg/m^2 ^and 800 mg/m^2^. No significant differences (p > 0.05) in mortality among the substrates were observed, indicating the effectiveness of chlorfenapyr on all the test substrates. From the results it can be inferred that a dose of 400 mg/m^2 ^chlorfenapyr was effective up to 24 weeks against *An. culicifacies *and up to 34 weeks against *An. stephensi*.

### Cross-resistance pattern to other insecticides

Laboratory and field populations of both *An. culicifacies *and *An. stephensi *with variable levels of resistance to DDT, malathion, bendiocarb and deltamethrin showed 100% mortality when exposed to 5.0% chlorfenapyr-treated papers indicating absence of cross resistance between chlorfenapyr and other insecticides (Table 2).

**Table 2:** Results of insecticide susceptibility tests on insecticide-susceptible (Sonepat and Nadiad) and -resistant (Goa) strains of *An. stephens i *and field-collected strains of *An. culicifacies *from Chhattisgarh and Gujarat states.

Species	Insecticides					Control		
	
	DDT 4.0%	Malathion 5.0%	Bendiocarb (0.1%)	Deltamethrin 0.05%	Chlorfenapyr 5.0%	OC	OP	PY
**Susceptible strains**								

*An. stephensi *(Sonepat)	98.3 ± 2.3* (57)	100 (48)	100 (102)	100 (68)	100 (169)	0 (35)	0 (15)	4.7 (21)

*An. stephensi *(Nadiad )	95.9 ± 2.8* (50)	98.0 ± 2.7* (48)	100 (30)	100 (49)	100 (125)	0 (16)	0 (45)	0 (17)

**Resistant strain**								

*An. stephensi *(Goa)	10.3 ± 5.1* (77)	26.2 ± 5.9* (46)	23.4 ± 2.1* (94)	84.9 ± 3.5* (47)	100 (116)	0 (15)	0 (36)	0 (15)

**Field collected strain - Raipur**								

*An. culicifacies*	4.2 ± 2.1* (120)	73.3 ± 3.9* (116)	80 ± 3.6* (30)	78.2 ± 2.5* (124)	100 (211)	0 (48)	0 (50)	0 (18)

**Field collected strain - Panchmahals**								

*An. culicifacies*	6.4 ± 2.6* (140)	30.1 ± 3.2* (123)	93.7 ± 2.7* (80)	43.1 ± 3.1* (130)	100 (60)	0 (21)	0 (39)	0 (20)

**Field collected strain - Vadodara**								

*An. culicifacies*	11.6 ± 2.9* (120)	41.1 ± 3.6* (124)	93.7 ± 3.2* (80)	59.2 ± 3.4* (130)	100 (60)	0 (20)	0 (40)	0 (20)

* - % Mortality + SE Figures in parentheses indicate number of mosquitoes exposed; OC: Organochlorine; OP: Organophosphate; PY: Pyrethroid; WHO criteria of adult susceptibility- susceptible- mortality between 98% and 100%; verification required-mortality between 97% and 80%, and resistant-mortality lower than 80%.

### Synergism/antagonism to PBO

**Figure 1:**
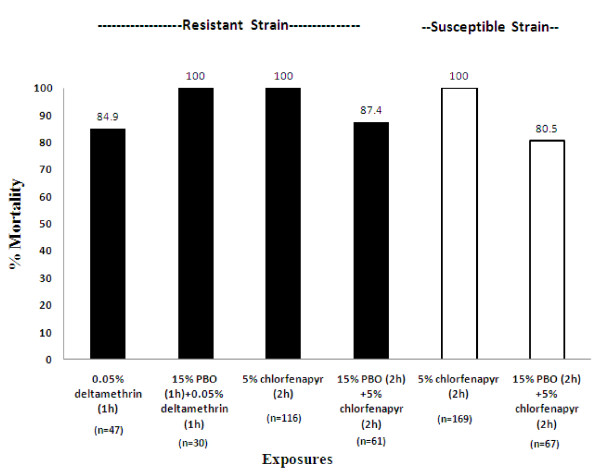
**Potentiation studies on susceptible (Sonepat) and resistant (Goa) strains of *An. stephensi***.

Exposure of insecticide-resistant *An. stephensi *(Goa strain) to deltamethrin resulted in 84.9% mortality while to PBO + deltamethrin the mortality increased to 100%. This indicates synergism and possible involvement of MFO-based metabolic resistance mechanism (Figure. 1). The LT50 and LT90 values for deltamethrin exposures alone were 20.3 min (95% FL = 17.522.9) and 35.4 min (95% FL = 29.4-40.5) respectively; while for PBO + deltamethrin exposure these were 16.9 min (95% FL = 16.06-17.76) and 21.2 min (95% FL = 20.09-23.06) respectively. These results support the observed synergism in toxicity to deltamethrin. The observed synergistic indices between deltamethrin alone and PBO + deltamethrin were 1.2 (LT50) and 1.7 (LT90) in insecticide-resistant *An. stephensi *(Goa strain).

Insecticide-resistant *An. stephensi *(Goa strain) registered 100% mortality against chlorfenapyr alone while it decreased to 87.4% when exposed to PBO + chlorfenapyr whereas mortality in insecticide-susceptible *An. stephensi *(Sonepat strain) was 100% when exposed to chlorfenapyr alone and decreased to 80.5% when exposed to PBO + chlorfenapyr, suggesting antagonistic effect of PBO (Figure. 1).

## Discussion

Laboratory evaluation of chlorfenapyr was carried out to determine the diagnostic dose to discriminate susceptible from resistant ones in mosquito populations. Studies were undertaken by spraying chlorfenapyr at different doses (12.5 to 800 mg a.i./m^2^) on different substrates to determine the persistence and effective dose of application for indoor sprays. Insecticide resistance pattern was determined using diagnostic dose on multiple insecticide-resistant laboratory-reared and field-collected mosquitoes.

A dose of 5.0% chlorfenapyr was found to be an effective diagnostic concentration for insecticide susceptibility tests. In India, as elsewhere in Asia, dwellings in rural areas present a wide variety of substrate materials. In laboratory conditions, persistence of different doses of chlorfenapyr on five different fabricated substrates was assessed by cone bioassays with susceptible strains of *An. stephensi *and *An. culicifacies *A up to 34 weeks. An application dose of 400 mg/m^2 ^was found effective on all the substrates, registering mortality of >80% up to 24 weeks in *An. culicifacies *A and up to 34 weeks in *An. stephensi.*

Knowledge of cross-resistance pattern conferring resistance to a particular candidate insecticide molecule is necessary to formulate strategies for resistance management [17] and to suggest effective and vector specific control methods for optimization of the efforts. The data on cross-resistance studies with field-collected DDT-malathion-deltamethrin-resistant *An. culicifacies *and laboratory-reared DDT-malathion-resistant/deltamethrin-tolerant *An. stephensi *(Goa strain) registered 100% mortality to chlorfenapyr, indicating absence of cross-resistance between chlorfenapyr and other insecticides. The findings of the present study has opened up the possibility of using chlorfenapyr as a potent candidate insecticide for malaria vector control in areas with multiple insecticide-resistant malaria vectors as suggested for the management of pyrethroid-resistant *An. gambiae *[18]; pyrethroid-resistant *An. gambiae *and *Cx. quinquefasciatus *in Benin [19]. Further, the reported mode of action of chlorfenapyr is through disruption of oxidative phosphorylation in the mitochondria rather than to target neural receptors, and absence of cross resistance was anticipated to the generally used insecticides for vector control including pyrethroids [3,20].

Potentiation studies with susceptible strain of *An. stephensi *(Sonepat) and resistant *An. stephensi *(Goa) with PBO have shown antagonism to chlorfenapyr toxicity. Such observation was also reported in insecticide-susceptible *Ae. aegypti *by Paul *et al *[10]. Monooxygenases metabolize chlorfenapyr to its active toxic insecticide form [21], and was demonstrated in the LS-VL strain of *Tetranychus urticae*, where PBO antagonized 2.3-fold toxicity of chlorfenapyr [22]. Similarly, in the present study with deltamethrin tolerant *An. stephensi *(Goa strain), PBO antagonized 1.1-fold toxicity of chlorfenapyr. This could be due to the less availability of MFOs owing to pre-exposure to inhibitor PBO, causing lower conversion rates of pro-insecticide chlorfenapyr to its toxic form. On the contrary, PBO has shown synergism to deltamethrin toxicity indicating involvement of elevated levels of MFOs as a resistance mechanism. It can be further stated that elevated levels of MFOs in pyrethroid tolerant/resistant mosquitoes may facilitate relatively increased conversion of pro-insecticide chlorfenapyr to its active form for increased toxicity.

Chlorfenapyr is a novel broad-spectrum insecticide currently registered in 19 countries for the control of various insect and mite pests on cotton, ornamentals and a number of vegetable crops [23]. Chlorfenapyr is also suggested to be a good candidate insecticide for malaria vector control in areas with pyrethroid-resistant *An. gambiae *[18,19,24] and *An. funestus *[25]. The results of the present study have indicated that chlorfenapyr can be used effectively for vector control, and for the management of multiple insecticide-resistant malaria vector species including pyrethroid resistant vectors.

## Conclusion

In the present study, a concentration of 5.0% chlorfenapyr was found effective as diagnostic concentration with 2 h exposure and 48 h holding period to assess susceptibility levels in *An. stephensi *and *An. culicifacies *mosquito. Laboratory studies on persistence of different doses of chlorfenapyr on artificially fabricated substrates, namely mud, mud + lime, cement and cement + distemper and native wood registered >80% mortality on cone bioassay up to 24 weeks in *An. culicifacies *A and up to 34 weeks in *An. stephensi *at an application dose of 400 mg/m^2^. Absence of cross-resistance was observed against chlorfenapyr in laboratory-reared multiple insecticide-resistant strains of *An. stephensi *and field-collected multiple insecticide-resistant *An. culicifacies *mosquitoes. Further, it is reported that the pro-insecticide chlorfenapyr is activated to a toxic insecticide form by MFOs. Potentiation studies with serial exposure to PBO and deltamethrin exhibited synergism in pyrethroid-resistant strains, indicating an MFO mediated resistance mechanism, while in serial exposures to PBO and chlorfenapyr, antagonism to chlorfenapyr toxicity was observed, probably due to lower bioavailability of MFOs owing to their inhibition by PBO. Thus, this molecule could be a potent candidate insecticide for malaria vector control, in areas with multiple-insecticide resistant malaria vectors, particularly in areas with synthetic pyrethroid-resistant vectors.

## Competing interests

The authors declare that they have no competing interests.

## Authors' contributions

Conceived and designed the experiments: KR, APD. Performed the experiments: KR, TKB, PS, RMB, HCS. Analysed the data: KR, TKB, US. Wrote the paper: KR, TKB. Contributed to data interpretation and critically reviewed the paper: KR, TKB, US. Critically reviewed the manuscript: KR, APD. All authors read and approved the final manuscript.
